# Supplementary material for: Analysis of PPARγ Signaling Activity in Psoriasis
Source: Int J Mol Sci. 2021 Aug 10;22(16):8603. doi: 10.3390/ijms22168603 (PMC8395241; doi:10.3390/ijms22168603)
Supplement: Supplementary file 1 [file ijms-22-08603-s001.zip › Supplemental materials_Analysis of PPARg signaling activity in psoriasis/Pathway models/Models images and html files/Model of PPARG related pathways in psoriasis (short version)/125745.html]

TICAM2


# Protein TICAM2

|  |  |
| --- | --- |
| URN | urn:agi-llid:353376 |
| Total Entities | 0 |
| Connectivity | 227 |
| Name | TICAM2 |
| Description | toll-like receptor adaptor molecule 2 |
| Notes | TIRP is a Toll/interleukin-1 receptor (IL1R; MIM 147810) (TIR) domain-containing adaptor protein involved in Toll receptor signaling (see TLR4; MIM 603030).[supplied by OMIM, Apr 2004] |
| Primary Cell Localization | Cytoplasm |
| ObjectType | Protein |

---

|  |  |
| --- | --- |
| Pathway | Cortisol in Resolving Inflammation |
|  | Antiviral Signaling through Pattern Recognition Receptors |
|  | TLR4 Signaling in Leukocytes |
|  | T-Cell Independent B-Cell Activation |
|  | Toll-like Receptors in Sterile Inflammation |
|  | Necrosis |
|  | MC1R in anti-Inflammatory Signaling |
|  | Macrophage M1 Lineage |
|  | CD16/CD14 Proinflammatory Monocyte Activation |
|  | AXL Receptor Inhibits Macrophages and Dendritic Cells Function |
|  | Dendritic Cells Function in Atherosclerosis |
|  | Myocardial Infarction |
|  | MAVS in Antiviral Innate Immune Response in Myocarditis |
|  | Toll-like Receptors (TLR) in Antiviral Innate Immune Response |
|  | Dendritic Cell Activation in Systemic Lupus Erythematosis |
|  | Crohn's Disease |
|  | Dendritic Cell Dysfunction in Crohn's disease |
|  | Dendritic Cell Function in Ulcerative Colitis |
|  | Nicotine anti-Inflammatory Effect in Ulcerative Colitis |
|  | Club-Cell Role in Asthma |
|  | Antigen-Presenting Cell Role in Asthma |
|  | Mucin Production in Goblet Airway Epithelial Cells |
|  | Airway Epithelial Cell Dysfunction |
|  | Monocytes Function in Gout |
|  | TLR2 Induced Synovial Fibroblast Activation in Rheumatoid Arthritis |
|  | Toll-like Receptors (TLR) in Periodontitis |
|  | IL1 Receptor Antagonist (IL1RN) Deficiency (DIRA) |
|  | PAPA Syndrome |
|  | TNF Receptor Associated Periodic Syndrome |
|  | Mevalonate Kinase Deficiency |
|  | Blau Syndrome |
|  | Cryopyrin (NLPR3) Associated Periodic Syndromes |
|  | Familial Cold Autoinflammatory Syndrome 2 |
|  | Familial Mediterranean Fever |
|  | Majeed Syndrome |
|  | HMGB1 and IL1B in Neuroinflammation |
|  | HPV E6 and E7 Induced Disruption of TNFR/NF-kB Signaling in Keratinocytes |
|  | Alveolar Macrophages Dysfunction in COPD |
|  | Mucin Hyperproduction in Goblet and Mucous Cells |
|  | TLR4 -> NF-kB/IRF Expression Targets |
|  | TLR4 -> IRF Signaling |
|  | pathway 4 |
|  | Model of PPARG signaling in psoriasis |
|  | Model of PPARG related pathways in psoriasis (short version) |
|  | sars-cov no binding |
|  | Dendritic Cell Activation in COVID19 |
|  | 1\_Insufficient activation of immune response in the middle ear epithelium cells in otitis media |
|  | 3\_Role of alveolar macrophages in pulmonary tissue destruction in COPD |
|  | 1\_Th2 cell response in asthma |
|  | 1\_Defects in response to pathogens in the gut promotes inflammation in Crohn's disease |
|  | 2\_Intestine epithelial cells drive immunological responses in ulcerative colitis |
|  | 1\_Intestine epithelial cell dysfunction in ulcerative colitis |
|  | 1\_Antigen-presenting cells promote islet dysfunction and immune system activation in T1D |
|  | 2\_Neuron and Schwann cell death |
|  | Model of PPARG signaling in psoriais (tested) |

---

|  |  |
| --- | --- |
| Group | other partners for SARS |

---

|  |  |
| --- | --- |
| MedScan ID | 353376 |

---

|  |  |
| --- | --- |
| LocusLink ID | 353376 |
|  | 225471 |
|  | 364867 |

---

|  |  |
| --- | --- |
| Alias | TIRAP3a |
|  | OTTHUMP00000162868 |
|  | toll-like receptor adaptor molecule II |
|  | Toll-interleukin I receptor domain (TIR)-containing adaptor molecule (TICAM) 2 |
|  | TRAM |
|  | TICAM2 |
|  | TIRP |
|  | TIR domain-containing adaptor molecule 2 |
|  | TIRAP3 |
|  | MGC129877 |
|  | TRIF-related adaptor molecule TRAM |
|  | MGC107370 |
|  | MGC129876 |
|  | TIRAP3a short |
|  | NF-kappa-B-activating protein 502 |
|  | toll-interleukin 1 receptor (TIR) domain-containing adaptor protein |
|  | MyD88-4 |
|  | toll/interleukin-1 receptor domain-containing protein |
|  | TIR-containing adaptor molecule II |
|  | toll-like receptor adaptor protein 3 |
|  | OTTHUMP00000207407 |
|  | TRIF-related adaptor molecule |
|  | B430113A10 |
|  | toll-like receptor adaptor molecule 2 |
|  | TIR-containing adaptor molecule 2 |
|  | TICAM-2 |
|  | cytoplasmic adaptor |
|  | putative NF-kappa-B-activating protein 502 |
|  | toll/interleukin-1 receptor (TIR) domain-containing adapter protein |
|  | TIR domain-containing adapter molecule 2 |
|  | Trif |
|  | TIR domain-containing adapter protein |
|  | TRIF-related adapter molecule TRAM |
|  | TIR-containing adapter molecule II |
|  | TRIF-related adapter molecule |
|  | Toll-interleukin I receptor domain (TIR)-containing adapter molecule (TICAM) 2 |
|  | TIR-containing adapter molecule 2 |
|  | toll-like receptor adapter molecule 2 |
|  | toll-like receptor adapter molecule II |

---

|  |  |
| --- | --- |
| GO ID | 0006888 |
|  | 0007030 |
|  | 0007249 |
|  | 0002756 |
|  | 0035669 |
|  | 0035666 |
|  | 0097190 |
|  | 0071222 |
|  | 0006954 |
|  | 0045087 |
|  | 0006886 |
|  | 0070266 |
|  | 0034128 |
|  | 0043312 |
|  | 0006909 |
|  | 0043123 |
|  | 2000494 |
|  | 0034145 |
|  | 0034142 |
|  | 0030134 |
|  | 0005794 |
|  | 0042995 |
|  | 0031901 |
|  | 0005783 |
|  | 0005793 |
|  | 0010008 |
|  | 0031902 |
|  | 0001891 |
|  | 0005886 |
|  | 0030667 |
|  | 0005149 |
|  | 0005543 |
|  | 0046982 |
|  | 0051607 |
|  | 0002376 |
|  | 0071650 |
|  | 0034144 |
|  | 0071651 |
|  | 0032729 |
|  | 0032755 |
|  | 0043122 |
|  | 0001817 |
|  | 0045357 |
|  | 0070671 |
|  | 0007165 |
|  | 0005737 |
|  | 0005769 |
|  | 0005768 |
|  | 0045323 |
|  | 0005770 |
|  | 0016020 |
|  | 0005622 |
|  | 0034138 |
|  | 0008063 |
|  | 0004871 |
|  | 0002224 |
|  | 0004888 |
|  | 0002755 |
|  | 0034130 |
|  | 0034134 |
|  | 0031224 |

---

|  |  |
| --- | --- |
| KEGG ID | mmu:225471 |
|  | hsa:353376 |
|  | rno:364867 |

---

|  |  |
| --- | --- |
| Organism | Homo sapiens {Organism urn:agi-taxid:9606} |
|  | Mus musculus {Organism urn:agi-taxid:10090} |
|  | Rattus norvegicus {Organism urn:agi-taxid:10116} |
|  | Homo sapiens |
|  | Mus musculus |
|  | Rattus norvegicus |

---

|  |  |
| --- | --- |
| Mouse chromosome position | 18 |

---

|  |  |
| --- | --- |
| OMIM ID | 608321 |

---

|  |  |
| --- | --- |
| Rat chromosome position | 18q11 |

---

|  |  |
| --- | --- |
| Hugo ID | 21354 |
|  | HGNC:21354 |

---

|  |  |
| --- | --- |
| Human chromosome position | 5q22.3 |
|  | 5q23.1 |

---

|  |  |
| --- | --- |
| Swiss-Prot Accession | Q86XR7 |
|  | Q86XR7.1 |
|  | Q8BJQ4 |
|  | Q8BJQ4.1 |
|  | D3ZXR7 |
|  | Q3U996 |

---

|  |  |
| --- | --- |
| GenBank ID | NC\_000005 |
|  | NM\_021649 |
|  | NP\_067681 |
|  | AC010226 |
|  | CH471086 |
|  | EAW48960 |
|  | AB002444 |
|  | AB091054 |
|  | BAC98397 |
|  | AB097022 |
|  | BAC77375 |
|  | AB109098 |
|  | BAC98399 |
|  | AB446491 |
|  | BAG55268 |
|  | AY232653 |
|  | AAO74498 |
|  | AY304582 |
|  | AAQ97431 |
|  | BC109265 |
|  | AAI09266 |
|  | BC109266 |
|  | AAI09267 |
|  | BX956265 |
|  | CA441688 |
|  | CD638761 |
|  | DA092564 |
|  | DA899189 |
|  | DA992598 |
|  | Q86XR7 |
|  | NC\_000084 |
|  | NM\_173394 |
|  | NP\_775570 |
|  | AC127342 |
|  | CH466528 |
|  | EDL09980 |
|  | AB100441 |
|  | BAC98398 |
|  | AK054192 |
|  | AK080873 |
|  | BAC38055 |
|  | AK134103 |
|  | BAE22014 |
|  | AK151885 |
|  | BAE30771 |
|  | AY268050 |
|  | AAP29979 |
|  | AY275837 |
|  | AAP81749 |
|  | BC099933 |
|  | AAH99933 |
|  | Q8BJQ4 |
|  | NC\_005117 |
|  | NM\_001108890 |
|  | NP\_001102360 |
|  | XM\_006254690 |
|  | XP\_006254752 |
|  | AC\_000086 |
|  | AABR07031956 |
|  | AAHX01094883 |
|  | AAHX01094884 |
|  | CH473971 |
|  | EDM14388 |
|  | EDM14394 |
|  | AC\_000137 |
|  | XP\_005272044 |
|  | AMYH02012005 |
|  | AAHY01138318 |
|  | AAHY01138319 |
|  | XM\_006525868 |
|  | AABR06094987 |
|  | ABBA01069032 |
|  | XM\_005271987 |
|  | AC\_000040 |
|  | NC\_018916 |
|  | XP\_006525931 |
|  | NT\_034772 |
|  | NW\_001838952 |
|  | NW\_922751 |
|  | AC\_000048 |
|  | NT\_039674 |
|  | NW\_001030635 |
|  | NW\_047513 |
|  | NW\_001084736 |

---

|  |  |
| --- | --- |
| Swiss-Prot ID | TCAM2\_MOUSE |
|  | D3ZXR7\_RAT |
|  | TCAM2\_HUMAN |

---

|  |  |
| --- | --- |
| Cell Localization | Cytoplasm |
|  | Golgi apparatus |
|  | Cell membrane |
|  | Early endosome |
|  | Late endosome |
|  | Endoplasmic reticulum |
|  | Cell projection |
|  | Phagocytic cup |

---

|  |  |
| --- | --- |
| Ensembl ID | ENSG00000243414 |
|  | ENSP00000415139.2 |
|  | ENST00000427199.2 |
|  | ENSMUSG00000056130 |
|  | ENSMUSP00000066239.3 |
|  | ENSMUST00000070084.10 |
|  | ENSRNOG00000042070 |
|  | ENSRNOP00000056221.2 |
|  | ENSRNOT00000059466.2 |
|  | ENSRNOT00000059466 |
|  | ENSMUST00000070084 |
|  | ENST00000427199 |
|  | ENSRNOP00000056221 |
|  | ENSMUSP00000066239 |
|  | ENSP00000415139 |
|  | ENSMUST00000070084.9 |

---

|  |  |
| --- | --- |
| MGI ID | MGI:3040056 |
|  | 3040056 |

---

|  |  |
| --- | --- |
| RGD ID | 1308258 |

---

|  |  |
| --- | --- |
| Unigene ID | Rn.225789 |
|  | Mm.149280 |
|  | Hs.718838 |
|  | Hs.709120 |
|  | Mm.484659 |

---

|  |  |
| --- | --- |
| Homologene ID | 11014 |

---

|  |  |
| --- | --- |
| IPI ID | IPI00222444 |
|  | IPI00782769 |
|  | IPI00360266 |

---
